# Health-related research publications on religious mass gatherings of Muslims: a bibliometric analysis (1980–2020)

**DOI:** 10.1186/s40794-021-00158-y

**Published:** 2022-01-04

**Authors:** Waleed M. Sweileh

**Affiliations:** Department of Physiology, Pharmacology/Toxicology, College of Medicine and Health Sciences, An-Najah National University, Nablus, Palestine

**Keywords:** Islam, Travel medicine, Religious mass gatherings, Saudi Arabia, Bibliometrics

## Abstract

**Objective:**

Mass gatherings medicine is an emerging and important field at the national and international health security levels. The objective of the current study was to analyze research publications on religious mass gatherings of Muslims using bibliometric tools.

**Methods:**

Keywords related to religious mass gatherings of Muslims were used in Scopus database. The duration of the study was from January 01, 1980 to December 31, 2020. Examples of keywords used include *hajj*, *Umrah*, mass gatherings/Mecca or Makkah, mass gatherings/Karbala, pilgrim/Makkah or Mecca, and others. Bibliometric indicators and mapping were presented.

**Results:**

In total, 509 documents were retrieved. The average number of citations per article was 16.7 per document. Analysis of the retrieved documents indicated that (1) more than 90% of the retrieved documents were about the mass gatherings in Mecca/Makkah; (2) two-thirds of the retrieved documents were research articles; (3) a take-off phase in the number of publications was observed after 2008; (4) the retrieved documents were disseminated in a wide range of journals but specifically the ones in the fields of infectious diseases, public health, and travel medicine; (5) the retrieved documents were mainly published by scholars from Saudi Arabia with collaborative research ties with scholars in the US, France, the UK, and Australia; (6) Saudi Arabia contributed to more than half of the retrieved documents; and (7) four research themes were found: knowledge, attitude, and practices of pilgrims to Mecca/Makkah, vaccination, etiology of hospital admission among pilgrims, and epidemiology of various types of infectious diseases.

**Conclusions:**

Research on mass gatherings, specifically the *Hajj,* is emerging. Researchers from the Saudi Arabia dominated the field. Research collaboration between scholars in Saudi Arabia and scholars in low- and middle-income countries is needed and must be encouraged since these countries have weaker health systems to screen, monitor, and control the spread of infectious diseases because of the *Hajj* season.

## Background

A mass gathering is defined by the World Health Organization as “*a planned or spontaneous event where the number of people attending could strain the planning and response resources of the community or country hosting the event*” [1]. Mass gatherings medicine gained a lot of global attention after the first International Conference in Mass Gatherings Medicine in October 2010 in Saudi Arabia where *The Lancet Infectious Diseases Series* on mass gatherings was launched [2].

Major religious mass gatherings of Muslims include the travel of Muslims to Mecca/Makkah for *Hajj* or *Umrah*. *Hajj* is an annual Islamic religious gathering in which approximately 3–4 million Muslims from all over the world visit Mecca in Saudi Arabia for 2–3 weeks to perform one of the most important pillars of Islam [3]. The time of *Hajj* is changing based on the Western calendar because the Islamic year is 11 days lesser than the Western year. Muslims can also go to Mecca/Makkah to perform *Umrah*, a minor pilgrimage, at any time of the year. In both *Hajj* and *Umrah*, the huge mass gatherings in a small area, fluctuating weather, and the hostile desert of Saudi Arabia expose pilgrims to health risks [4–6].

There are other important mass gatherings of Muslims. The Shiite (Shia) Muslims gather in Karbala (Iraq) at the *“Arba’een”* event. The *Arba’een* gatherings occur at the end of the 40-day mourning period following *Ashura*, the religious ritual for the commemoration of the martyrdom of Imam, *Husayn ibn Ali*, the grandson of Prophet Mohammad. In 2016, approximately 25 million people visited Karbala with one-fifth being visitors from external countries such as Iran and Pakistan [7, 8]. The “*Arba’een*” gathering in Iraq is now the largest annual public gathering in a single place worldwide [9]. A third important mass gathering event for Muslims is the *Grand Magal* of Touba, which is an annual 1-day Muslim religious event that takes place in Touba (Senegal). During this event, the population of Touba increases from approximately 1.5 million inhabitants to an estimated 4–5 million pilgrims coming from across Senegal and other African countries [10].

Bibliometric analysis is the use of mathematical and statistical tools to analyze the growth and developmental pattern of publications in a certain field [11–13]. Assessing and reviewing published research on religious mass gatherings is one potential indicator of the extent of national preparedness to public health emergencies and the extent of awareness of health hazards associated with religious mass gatherings. Furthermore, assessing publications on mass gatherings will provide policymakers with information needed for better health planning and management of these mass gatherings [14–19]. Governments of Islamic countries need to utilize information obtained from research analysis to implement health-related initiatives to contain the potential spread of certain infectious diseases due to religious mass gatherings. Finally, bibliometric analysis provides academics, researchers, and policy makers with information regarding research gaps to direct future funding and research activity in the field.

Bibliometric studies on mass gatherings or travel medicine-related literature have been previously published [20–22]. However, up to the author’s best knowledge, no bibliometric studies of literature on mass gatherings and travel to major Islamic religious events have been published. Therefore, the objective of this study was to provide readers, scholars, academics, and policymakers with a snapshot analysis of health-related publications on religious mass gatherings of Muslims.

## Methods

This was a cross-sectional analysis of documents published in peer-reviewed journals on the religious mass gatherings of Muslims.

### The database used in the search strategy

Scopus database was used to extract the relevant publications. The choice of Scopus was based on the idea that Scopus has a larger number of indexed journals and 100% inclusive of journals in Medline. Furthermore, Scopus has indexed journals in health, social, physical, and life sciences. This increases the chance of retrieving the maximum possible relevant publications. The advanced search function in Scopus allows for the development of a search strategy with an unlimited number of terms and the use of various types of Boolean operators. Finally, Scopus has many analytic functions that facilitate the export of data from Scopus to Microsoft Excel for analysis.

### Keywords used in the search strategy

The keywords used in the search strategy included:

1. Hajj, Umrah, Umra / Mekkah or Mecca or Saudi
2. Islamic/Muslim Pilgrim,
3. “Gathering” AND Mecca/Makkah, Touba, Karbala, Ashura, Arb’een
4. “Sri Petaling” AND Malaysia
5. “Religious gathering” AND Saudi or Iraq or Iran or Pakistan or Senegal.

The keywords were selected based on the literature review of mass gatherings of the major Muslim events [23, 24]. In the current study, to limit the retrieved documents to health publications, only documents published within the subject area of health were included.

### Inclusion and exclusion criteria

The search strategy was limited for the study period from January 01, 1980, until December 31, 2020. Only journal documents were included. Therefore, books and book chapters were excluded. No language restriction was imposed.

### Validation of the search strategy

The search strategy was validated using three different methods. First, two independent reviewers assured the absence of false-positive results in the top 30 cited articles. Second, the top active author, Memish, Z.A. produced a similar number of research articles compared to the number retrieved by the search strategy. Third, the top ten active journals in publishing the retrieved articles were relevant to the field. Finally, the top active country was Saudi Arabia that endorses the findings and the search strategy since Saudi Arabia is the major country involved in religious tourism to Mecca and Madinah.

### Data export and data management

The search strategy was implemented and the retrieved data were exported to Microsoft Excel as “csv” file. The data exported included information about the title/abstract of each document, author(s) name (s), country and institutional affiliation of the author(s), author keywords, the annual number of publications, citations, and journal names.

Data regarding annual growth, productivity, and citation analysis were presented in linear graphics or table format based on the exported information in Microsoft Excel. However, data regarding frequent author keywords, frequent terms in titles/abstracts, cross-country collaboration, and research networks of authors were visualized using the free online program VOSviewer [25]. In VOSviewer maps, nodes have different colors and sizes. The size of a node is proportional to the frequency of occurrence. The color of the node represents the relationship with other nodes having similar colors.

## Results

### General characteristics of the retrieved documents

The search strategy retrieved 509 documents related to religious mass gatherings of Muslims. The retrieved documents were of seven types, mainly research articles (*n* = 346, 68.0%). Other types of retrieved documents are shown in Table 1. The retrieved documents have 2811 author names, an average of 5.5 authors per document. Of the retrieved documents, 290 (57.0%) were published in open access journals. Of the 509 documents, 503 (98.8%) were published in English. The remaining documents (*n* = 6, 1.2%) were published in a non-English language. The retrieved documents received 8504 citations, an average of 16.7 citations per document. Of the 509 retrieved documents, 472 (92.7%) had the keyword “*Hajj*” in the title.

**Table 1:** Types of documents on religious mass gatherings of Muslims

Type of document	Frequency	% (***N*** = 509)
Article	346	68.0
Review	65	12.8
Letter	47	9.2
Editorial	30	5.9
Note	13	2.6
Short Survey	6	1.2
Conference Paper	2	0.4

### Growth trajectories of publications and citations

**Fig. 1:**
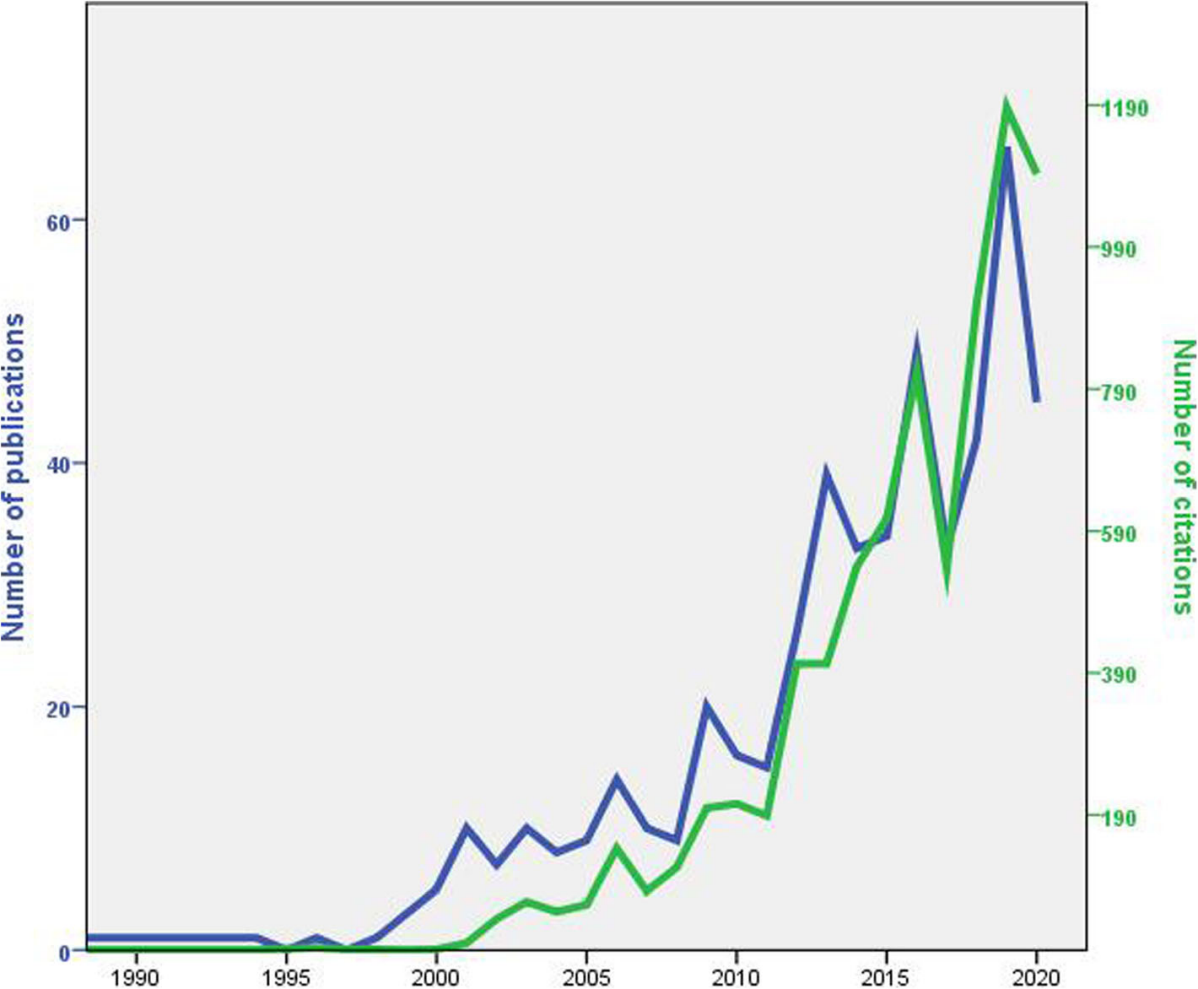
Growth of publications and citations on religious mass gatherings of Muslims

Figure 1 shows the growth trajectory of publications which can be organized into three distinct phases: an emergence phase (≤1998), a fermentation phase (1999–2008), and a take-off phase (2009–2020). The growth of citations during the study period showed a gradual increase that ultimately coincided with that of the number of publications.

### Core journals

The retrieved documents were published in 181 peer-reviewed scientific journals. The *Travel Medicine and Infectious Disease* journal (*n* = 57, 11.2%) ranked first in the number of publications. Table 2 shows the ten core journals. *The Lancet* journal ranked fifth in the core list. The majority of the journals in the core list were in the field of infectious diseases or public health or both.

**Table 2:** Core journals in publishing documents on religious mass gatherings of Muslims

Rank	Journal name	Frequency	% (***N*** = 509)
1	*Travel Medicine And Infectious Disease*	57	11.2
2	*Saudi Medical Journal*	28	5.5
3	*Journal Of Travel Medicine*	23	4.5
4	*International Journal Of Infectious Diseases*	20	3.9
5	*Lancet*	15	2.9
6	*Journal Of Epidemiology And Global Health*	12	2.4
7	*JMIR Public Health And Surveillance*	11	2.2
7	*Journal Of Infection And Public Health*	11	2.2
7	*Weekly Epidemiological Record Health Section Of The Secretariat Of The League Of Nations*	11	2.2
10	*Vaccine*	10	2.0

### Leading authors

In total, 2811 authors participated in publishing the retrieved documents. *Memish, Z.A.* (Saudi Arabia) ranked first (*n* = 107, 21.0%) in the number of publications. Table 3 shows the ten core researchers in the field. The core researchers were from Saudi Arabia, France, and Australia.

**Table 3:** Core countries in publishing documents on religious mass gatherings of Muslims

Rank	Country of origin	Frequency	% (***N*** = 509)
1	Saudi Arabia	278	54.6
2	United States	77	15.1
3	France	71	13.9
4	United Kingdom	62	12.2
5	Australia	54	10.6
6	Iran	41	8.1
7	Egypt	27	5.3
8	Malaysia	23	4.5
9	Canada	11	2.2
9	India	11	2.2
9	Pakistan	11	2.2
9	Viet Nam	11	2.2

### Leading countries

Authors from 61 different countries participated in publishing the retrieved documents. Saudi Arabia was the indisputable leader in this field, publishing more than half (*n* = 278, 54.6%) of the retrieved documents. Table 4 shows the ten core countries in the number of publications. The list of core countries included the US, France, the UK, Australia, Canada, and India with large Muslim communities. At the institutional level, the *Ministry of Health* of Saudi Arabia ranked first in the number of publications with 146 (28.7%) documents.

**Table 4:** Core researchers in publishing documents on religious mass gatherings of Muslims

Rank	Author name	Country of origin	Frequency	% (***N*** = 509)
1	Memish, Z.A.	Saudi Arabia	107	21.0
2	Gautret, P.	France	60	11.8
3	Rashid, H.	Australia	43	8.4
3	Yezli, S.	Saudi Arabia	43	8.4
5	Booy, R.	Australia	37	7.3
6	Parola, P.	France	35	6.9
7	Benkouiten, S.	France	26	5.1
8	Brouqui, P.	France	25	4.9
9	Al-Tawfiq, J.A.	Saudi Arabia	22	4.3
9	Alotaibi, B.	Saudi Arabia	22	4.3

### Networks of author collaboration

**Fig. 2:**
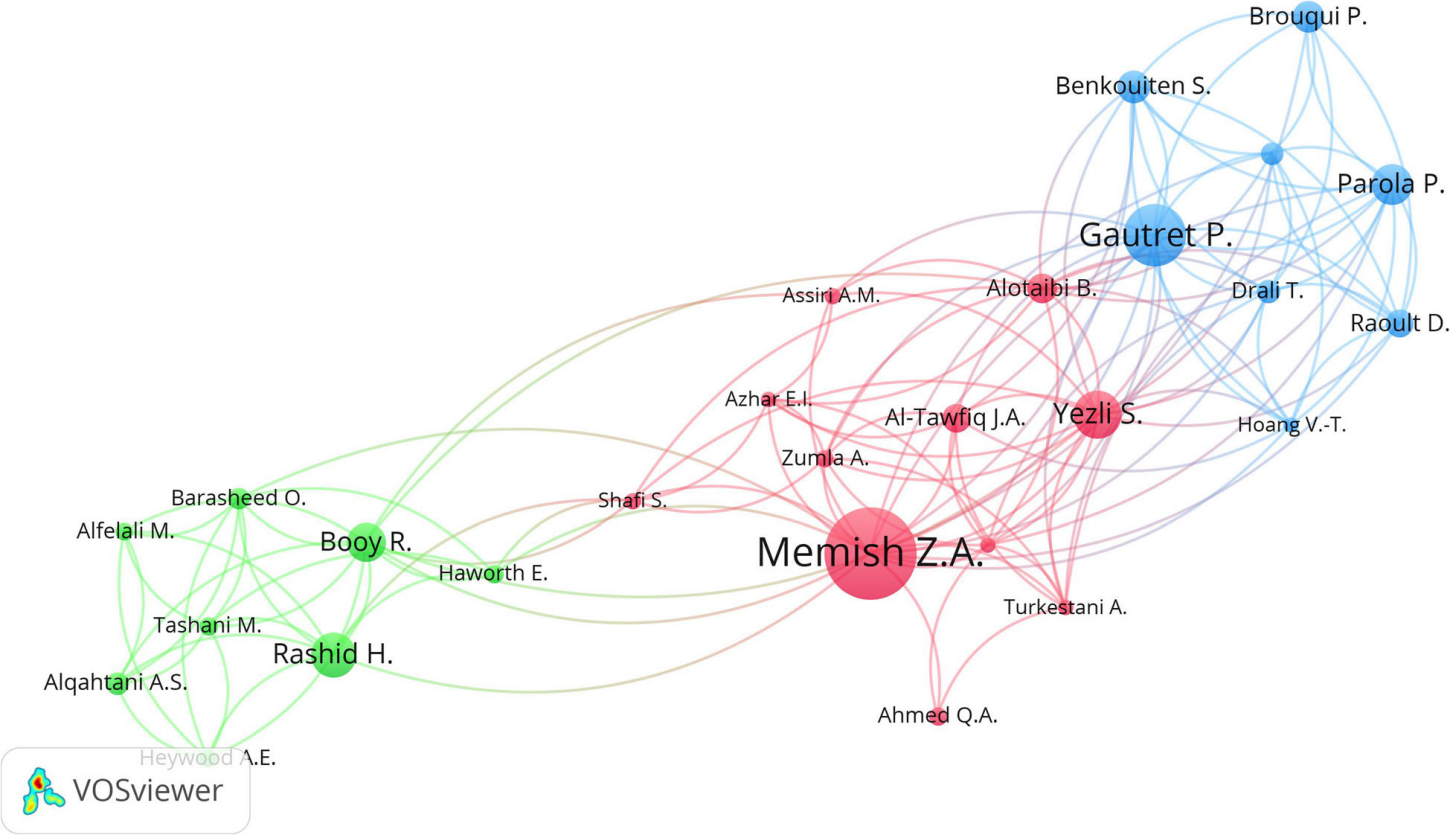
Collaborative research networks between researchers with a minimum number of publications of 10 documents. The number of researchers on the map was 27. The map has three clusters with three different colors. Each cluster represents a group of closely related researchers

Figure 2 is a network visualization map of authors with a minimum of 10 publications. The map included 27 authors. The red cluster included 11 authors and represented Saudi researchers led by *Memish, Z.A*. The Saudi cluster existed in the center with collaborative ties with the two remaining clusters. The blue cluster represented French researchers while the green cluster represented Australian researchers.

### Networks of international research collaboration

**Fig. 3:**
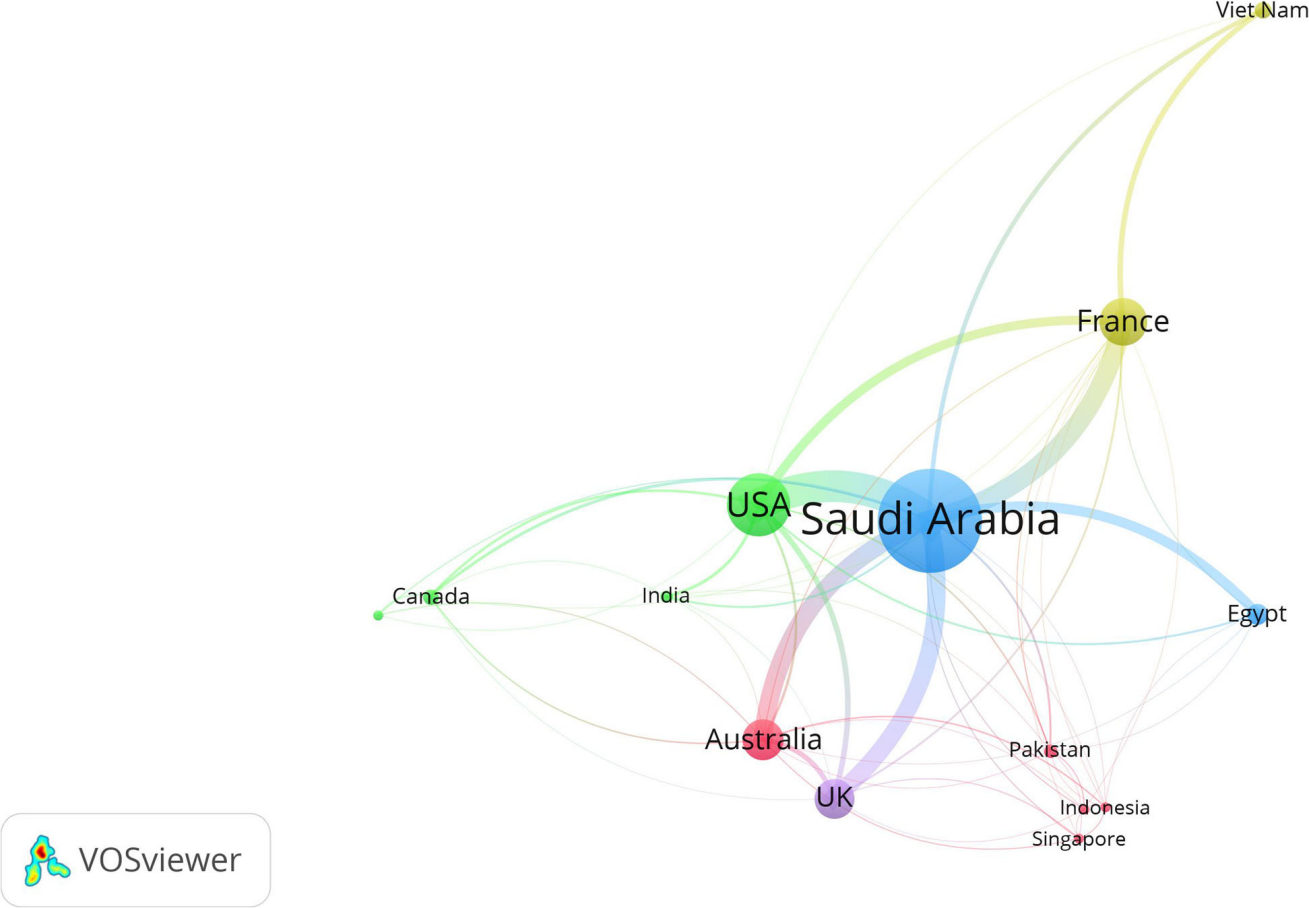
Collaborative research networks between countries with 10 or more publications. Only countries that exist in research networks were mapped. The thickness of the connecting line between two countries is proportional to the strength of research collaboration

Figure 3 is a network visualization map of countries with a minimum of 10 publications (*n* = 14). The map shows Saudi Arabia in the center of the map with collaborative ties with most countries on the map. The strongest international research collaboration was between Saudi Arabia and the US, followed by Saudi Arabia and France, Saudi Arabia and Australia, and Saudi Arabia and the UK.

### Co-occurrence analysis of author keywords (important topics)

**Fig. 4:**
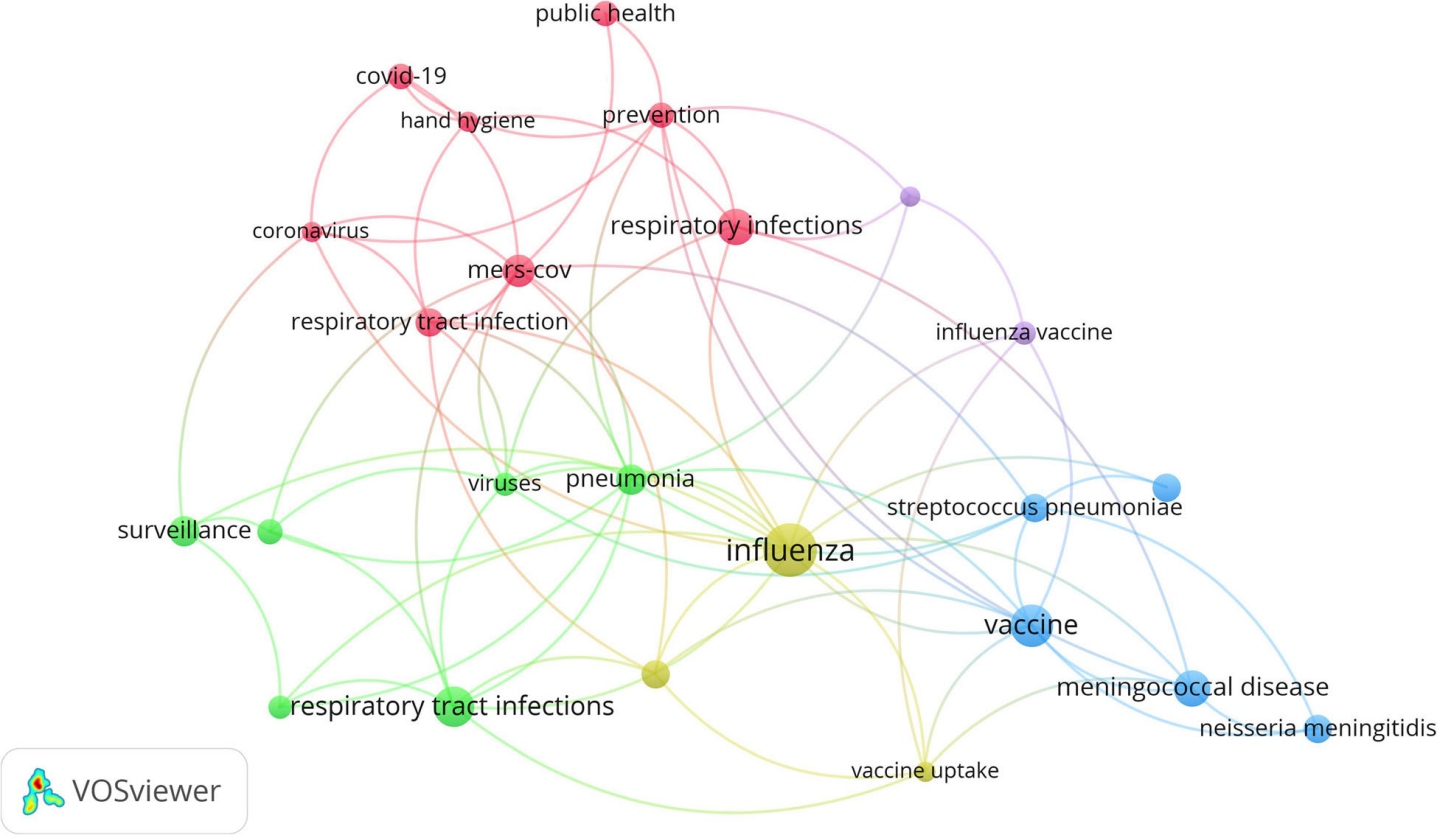
Network visualization map of author keywords with a minimum of five or more. Keywords such as “mass gatherings” and Hajj were excluded to expose other keywords. The map has 24 keywords. The size of the node is proportional to the number of occurrences in the retrieved documents and represents an important topic

Figure 4 shows a co-occurrence analysis of author keywords with a minimum occurrence of five. The analysis excluded keywords related to “mass gatherings”. Important keywords in the map included vaccination, respiratory tract infections, influenza, coronavirus, prevention, and knowledge.

### Most frequent terms in titles/abstracts (major research themes)

**Fig. 5:**
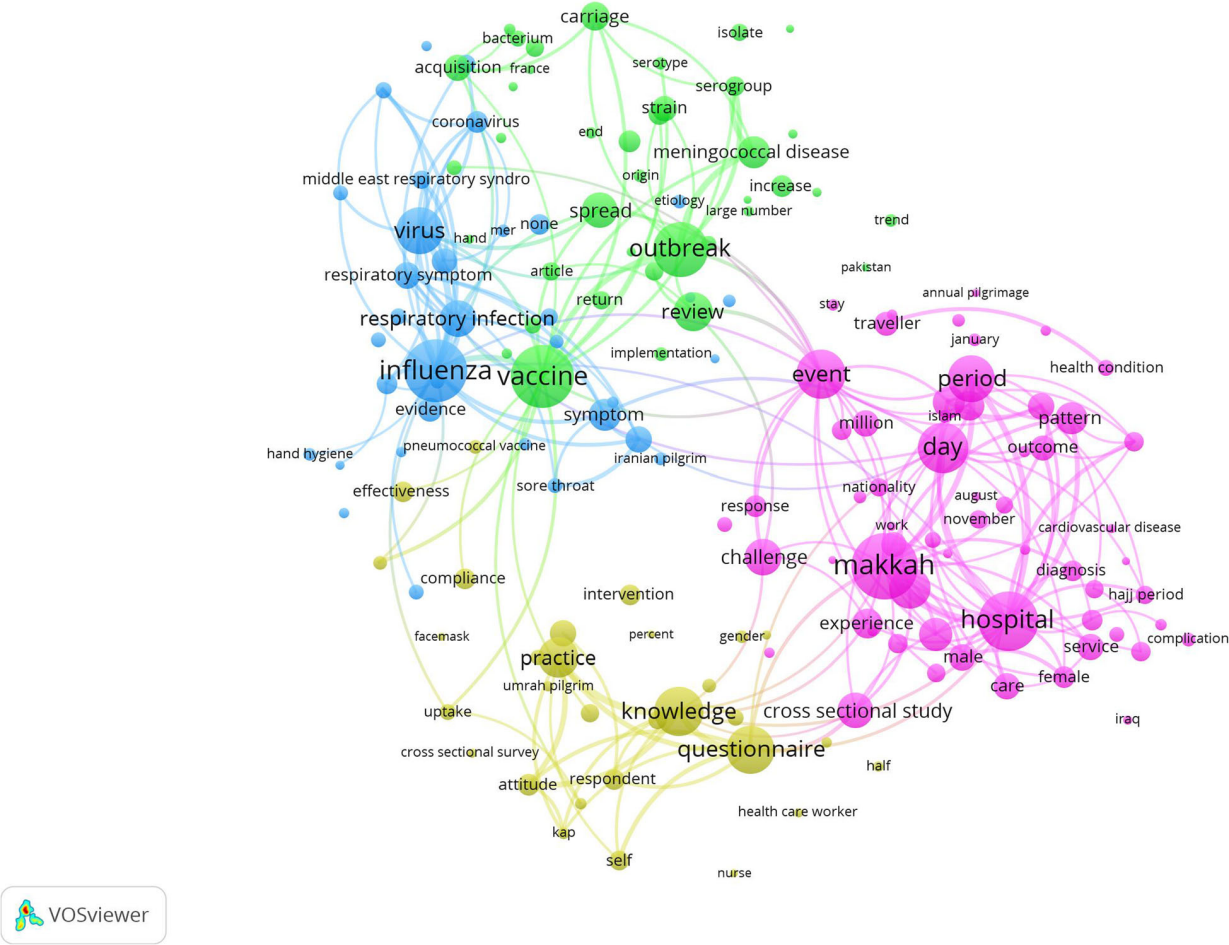
Network visualization map of terms in the titles/abstracts with a minimum occurrence of five or more. Nodes having similar color are closely related and represent a separate cluster. Each cluster represents a general research theme present in the retrieved documents

Network visualization map of terms in titles and abstracts with a minimum occurrence of five were mapped resulting in four clusters (research themes) (Fig. 5). The clusters were as follows:

1. The yellow cluster represents surveys and questionnaire-based studies on knowledge, attitude, and practices of pilgrims to Mecca/Makkah
2. The green cluster represents studies on vaccine-preventable outbreaks of meningitis, pneumonia, and other infections
3. The red cluster represents studies on etiology and epidemiology of hospital admission among pilgrims in Mecca/Makkah
4. The blue cluster represents studies on prevention, symptoms, and epidemiology of various viral infectious diseases

### Disciplines underlying the foundations of the field

**Fig. 6:**
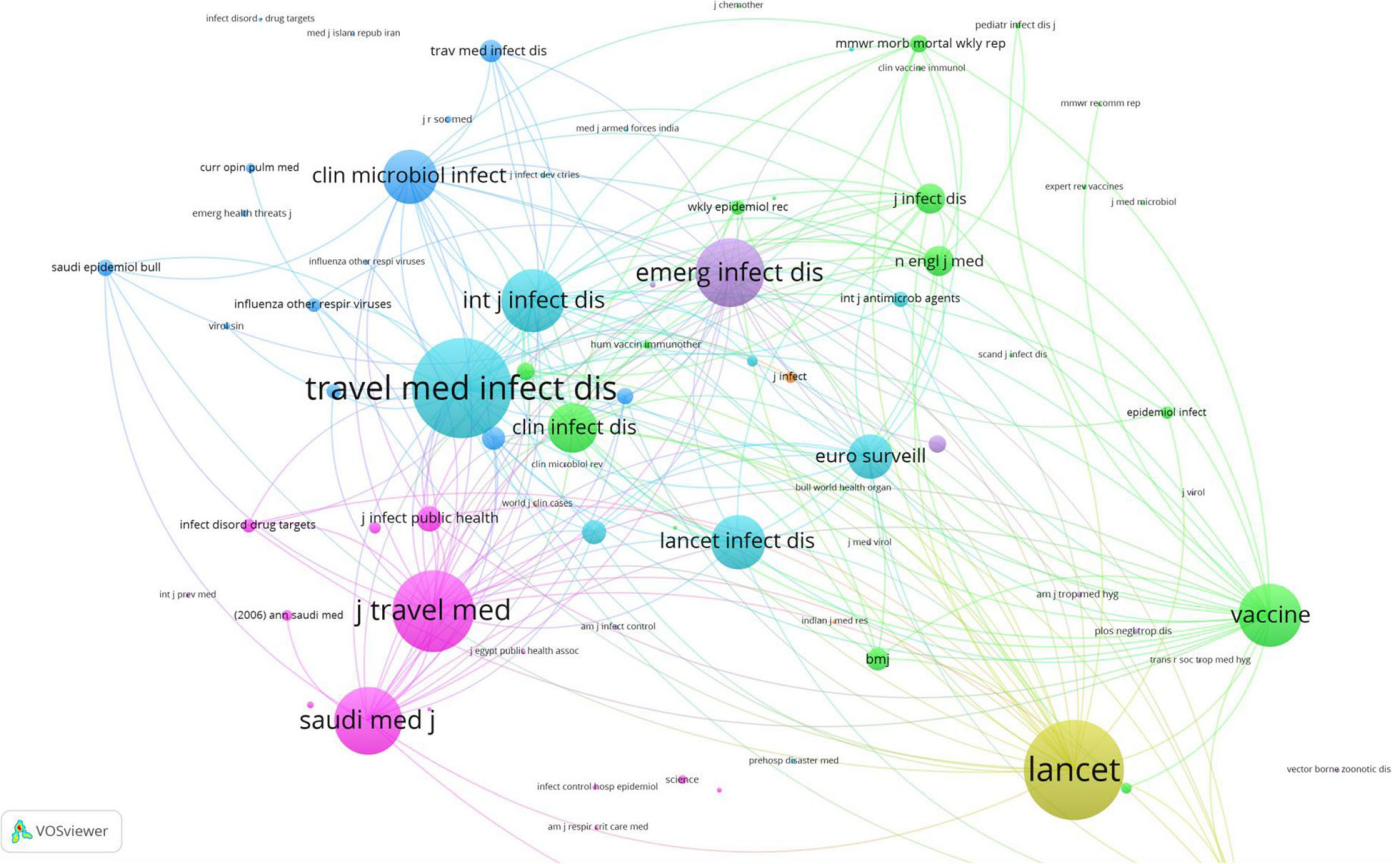
A map of journal co-citation analysis (*n* = 105 journals). A node with a large size represents journals that are most frequently co-cited by other journals with the same node color. The subject area of the largest nodes are the underlying fields of the retrieved documents (religious mass gatherings of Muslims)

Figure 6 is a co-citation analysis of journals with a minimum citation of 10 (*n* = 105 journals). The node size represents journals that were most commonly co-cited by journals having similar node colors. The subject area(s) of the journals with the largest node size represents disciplines underlying the field. In the current study, the field of mass gatherings of Muslims was formulated by general medicine (The Lancet, Saudi Medical Journal), infectious diseases/public health (Vaccine, Journal of Travel Medicine, Travel Medicine and Infectious Diseases), and infectious diseases (International Journal of Infectious Diseases, Emerging Infectious Diseases).

## Discussion

The current study provided a bibliometric overview of health-related publications on religious mass gatherings of Muslims. Below is a summary of the major findings of the current study. First, research on mass gatherings of Muslims focused entirely on the one that happened annually in Saudi Arabia, the *Hajj* season. Second, the last decade witnessed an increase in the number of health-related publications on mass gatherings of Muslims. However, given the annual nature of the *Hajj* season and the geographic distribution of the participants, the number of retrieved research articles was relatively small. Third, research investigation on health risks of religious mass gatherings was confined to scholars from Saudi Arabia and collaborating scholars from countries with Muslim communities or scholars interested in the public health impact of mass gatherings.

As a mass gathering of millions of people from the whole world, *Hajj* can facilitate the transmission of infectious diseases leading to a global public health dilemma [26–30]. A study indicated that more than 35% of the COVID-19 cases in Malaysia were directly linked to the “Sri Petaling” mass gathering attended by more than 19,000 Muslims from Malaysia, India, South Korea, Brunei, China, Japan, and Thailand [31]. That is why the government of Islamic countries such as Saudi Arabia imposed strict regulations on the numbers and age of people allowed to perform the pilgrimage to Mecca/Makkah in the last 2 years during the COVID-19 pandemic [32].

In the current study, the take-off phase in the number of publications was noticed after 2008. The steady rapid increase after 2008 coincides with several events that called for attention to the health risks and potential disasters of mass gatherings. The first International Conference on Mass gatherings medicine was held in Saudi Arabia in 2010 and led to the formation of a coalition of experts from the WHO and global public health experts to develop safety guidelines at mass gathering events [33, 34]. Several infectious disease outbreaks were recorded in the past two decades including coronavirus, Ebola, Influenza, Zika, and others, which forced the Saudi government to impose various types of restrictions, screening, and surveillance on pilgrims from affected areas. Infectious disease outbreaks in any country might affect the *Hajj* season because people from more than 100 different countries attend the *Hajj* season increasing the risk of the spread of infectious diseases. The recent emerging of research on mass gatherings was confirmed by a study on the emergence of literature on mass gatherings [22].

The current study indicated that the core journals on religious mass gatherings of Muslims included ones with high impact factors like *The Lancet*. This indicates that religious mass gathering, specifically the *Hajj* event, has a global serious health impact. The core journals included three journals, the *Saudi Medical Journal*, the *Journal of Epidemiology and Global Health*, and the *Journal of Infection and Public Health* that are affiliated with Saudi Institutions. This is expected given that Saudi Arabia is the major contributor to this field and that the major religious mass gathering is organized and monitored by the Saudi government. The extensive involvement of Saudi authors in research about religious mass gatherings was also noted in the number of publications contributed by Saudi Arabia. The finding that the US ranked second could be explained by the fact that certain Saudi researchers who are involved in research on mass gatherings have double institutional affiliations, in Saudi Arabia and the US. The author’s network map showed well-established research groups in France and Australia who are well connected with research groups in Saudi Arabia. The international research collaboration between scholars in Saudi Arabia and scholars in Australia and France has a positive impact on the ranking of these two countries in the core list.

The current study showed that one major research theme in the retrieved documents was the etiology and epidemiology of hospital admission of pilgrims during the *Hajj* season. A wide range of communicable and non-communicable diseases has been reported as potential causes of hospital admission during the *Hajj* season. Such causes include meningococcal diseases, respiratory tract infections including pneumonia, infectious diarrhea, skin infections, malaria, and emerging infections such as H1N1 and coronavirus [35, 36]. Non-communicable diseases include heat injury, surgery, and exacerbating pre-existing chronic conditions [37]. A systematic review study on infectious diseases and preventive measures during *Hajj* indicated that the most frequent diseases in *Hajj* were respiratory infections and the emerging and re-emerging diseases, such as Severe Acute Respiratory Syndrome (SARS), Middle East Respiratory Syndrome due to coronaviruses (MERS-CoV), and Ebola which imposed many stresses on the pilgrims and health policy managers [36]. A second systematic review study on the risks threatening the health of people participating in mass gatherings indicated the presence of more than forty health-related risks that can be classified into five domains: environmental risks, individual risks, psychological risks, public health risks, and management risks [38].

The current study has a few limitations. The presence of false-positive and false-negative results is a possibility despite that the author did his best to minimize this type of error. Furthermore, the fact that we used Scopus to retrieve documents might have led to the loss of certain documents published in local un-indexed journals in Arab or Islamic countries.

## Conclusions

The current study is the first to provide a bibliometric overview of research publications on religious mass gatherings of Muslims. The bulk of the retrieved documents were published in the last decade. The current study showed that more than 90% of the retrieved documents were *Hajj* with clear dominance of researchers, institutions, and journals in Saudi Arabia. Analysis also showed dominance of research topics related to infectious diseases. *Hajj* provides an opportunity for international health bodies and governments to monitor national and global health security by prevention of outbreaks of any particular infectious diseases when pilgrims return home [6]. Future research should focus on other religious mass gatherings in Iraq and other countries as well. Mass gatherings medicine needs to be introduced into medical education in Islamic countries where religious gatherings occur daily in prayers and pilgrimage to *Hajj* or *Umrah*.

## Data Availability

all data present in this article can be retrieved from Scopus using keywords listed in the methodology.
